# Early and intermediate outcomes for surgical management of infective endocarditis

**DOI:** 10.1186/s13019-019-1029-1

**Published:** 2019-12-03

**Authors:** Lindsay Volk, Nina Verghis, Antonio Chiricolo, Hirohisa Ikegami, Leonard Y. Lee, Anthony Lemaire

**Affiliations:** Division of Cardiothoracic Surgery, Department of Surgery, RUTGERS-Robert Wood Johnson Medical School, 125 Paterson Street, New Brunswick, NJ 08903 USA

**Keywords:** Endocarditis, Intravenous drug use, Valve replacement

## Abstract

**Objective:**

The treatment of active infective endocarditis (IE) presents a clinical dilemma with uncertain outcomes. This study sets out to determine the early and intermediate outcomes of patients treated surgically for active IE at an academic medical center.

**Methods:**

A retrospective chart review was conducted to identify patients who underwent surgical intervention for IE at our institution from July 1st, 2011 to June 30th, 2018. In-patient records were examined to determine etiology of disease, surgical intervention type, postoperative complications, length of stay (LOS), 30-day in-hospital mortality, and 1-year survival.

**Results:**

Twenty-five patients underwent surgical intervention for active IE. The average age of the patients was 47 ± 14 years old and most of the patients were male (*N* = 15). The majority of the patients had the mitral valve replaced (*N* = 10), with the remaining patients having tricuspid (*N* = 8) and aortic (*N* = 7) valve replacements. The etiology varied and included intravenous drug use (IVDU), and presence of transvenous catheters. The 30-day in-hospital mortality was 4% with 1 patient death and the 1-year survival was 80%. The average LOS was 27 days ±15 and the longest LOS was 65 days.

**Conclusions:**

Surgical management of IE can be difficult and challenging however mortality can be minimized with acceptable morbidity. The most common complication was CVA. The average LOS is longer than traditional adult cardiac surgery procedures and the recurrence rate of valvular infection is not minimal especially if the underlying etiology is IVDU.

## Introduction

Infective endocarditis (IE) is a rare disease, but its impact is significant [1]. It affects 3 to 10 per 100,000 per year in the population at large and the studies suggest that the incidence is rising [2]. Despite recent advances in treatment, IE remains a life-threatening disease with significant morbidity and mortality [3, 4]. The rise of antibiotic resistance against the main causative organisms has made medical management increasingly difficult [4]. As a complement to the medical management of IE, surgery remains a treatment option for some of the patients with IE.

Surgical intervention is warranted in IE for a variety of indications including unsuccessful medical therapy, involvement of a prosthetic valve, recurrent embolization, or embolization with residual large vegetations [3]. Over the past 10 years, there is evidence to suggest the importance of surgery as a treatment option in this population [3]. The surgical treatment of active infectious endocarditis unfortunately has an associated high mortality and morbidity [4]. Consequently, there are controversies regarding the timing of surgery, either in the active phase of IE or after the completion of the antibiotic therapy [5, 6]. The purpose of the study is to review the institutional experience of the surgical management of IE at an academic institution.

## Methods

A retrospective chart review was conducted to identify patients who underwent surgical intervention for IE at our institution from July 1st, 2011 to June 30th, 2018. Patient were identified using procedural and diagnosis codes for valve replacement or repair for infective endocarditis respectively. In-patient records were then examined to determine etiology of disease and surgical intervention type. Charts were also reviewed to determine postoperative complications, length of stay (LOS), 30-day in-hospital mortality, and 1-year survival. Postoperative complications were collected as free-text variables and later grouped by the authors. Complication groups included cerebral vascular accident (CVA), acute kidney injury, and cardiac complications. Continuous variables are reported as means with standard deviations. All other variables are reported as frequencies.

## Results

We identified 25 patients who underwent surgical intervention for active IE during the study period. The average age of the patients was 47 ± 14 years old and most of the patients were male (*N* = 15). The etiology varied and the most common indications were intravenous drug use (IVDU) and presence of transvenous catheters. The length of hospital stay prior to surgery was 9.4 ± 8.5 days. A total of 4 patients (16%) underwent emergent surgery, and the remainder underwent surgeries that were scheduled in the operating room as non-emergent.

**Fig. 1:**
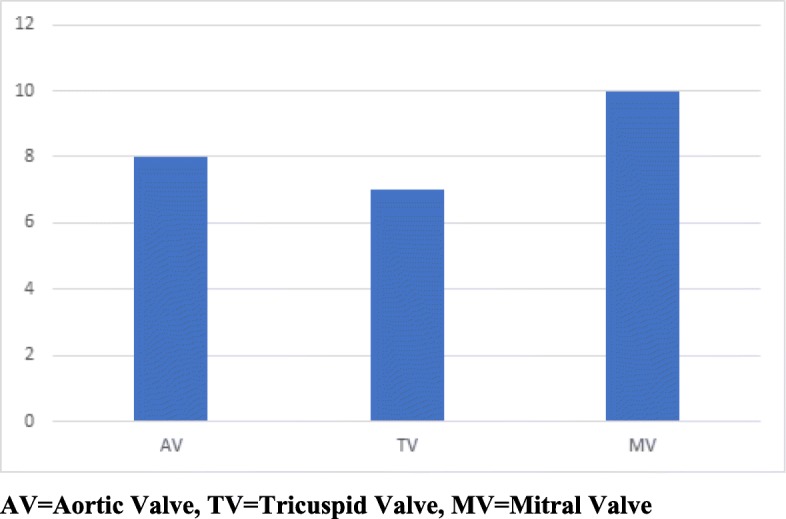
Distribution of involved valves. AV = Aortic Valve, TV = Tricuspid Valve, MV = Mitral Valve

The preoperative factors are detailed in Table 1. The majority of the patients had the mitral valve replaced (*N* = 10), with the remaining patients having tricuspid (*N* = 8) and aortic (*N* = 7) valve replacements (Fig. 1). None of the patients in the study had valvular repair but rather valve replacement was performed. In addition, pericardial tissue valves rather than mechanical valves were used in all of the cases. Specifically, all the patients received either a porcine or bovine valve. Post operatively, four patients (16%) developed cerebrovascular accidents and 1 patient (4%) required a craniotomy. The patient required the craniotomy because of cerebral edema and was found intraoperatively to have a small hematoma and cerebral swelling. Five of the 25 patients (20%) returned with recurrent IE and two patients (8%) required redo valvular replacements. The cause of the recurrence was resumed IVDU in all 5 cases. Three of the patients with recurrent disease were treated with IV antibiotics as they were deemed too high risk for redo surgery. The 30-day in-hospital mortality was 4% with 1 patient death while in the hospital and the 1-year survival was 80%. The average postoperative LOS was 17.8 ± 12.6 and average total LOS was 27 days ±15. The longest total LOS was 65 days. None of the patients developed bleeding requiring redo surgery or mediastinitis (Table 2).

**Table 1:** Summary of preoperative patient factors

Demographics	
Age	47 ± 14
Male	15 (60%)
Urgent surgery	4 (16%)
Length of stay prior to surgery	9.4 ± 8.5

**Table 2:** Summary of postoperative outcomes

Study variables	
Post-operative length of stay	17.8 ± 12.6
Total length of stay	27.2 ± 16.0
30-day in-hospital mortality	0 (0%)
In-hospital mortality	1 (4%)
CVA	4 (16%)
1-year survival	20 (80%)

## Discussion

The data from our study shows that surgical management of IE can be effective with limited although serious complications. The patients in our study had only a 4% 30-day mortality and an 80% survival at 1 year. The death of the patients after the initial 30 days was related to the development of sepsis (*N* = 2), complications related to stroke (*N* = 1), and the development of multisystem organ failure (*N* = 2). The morbidity of the procedure is seen with 16% of the patients developing a CVA. Fortunately, none of the patients developed infectious complications nor had bleeding issues. One of the key factors that continues to be a problem in the management of patients with IE is the recurrence rate of endocarditis in these patients. There was a 20% recurrence rate (*N* = 5) in the patients with 2 of the 5 patients undergoing redo-valvular surgery.

The rate of recurrence in this study was markedly higher than in other studies. A study by Jault et al., found a recurrence rate of prosthetic valve endocarditis was up to 7% within a 6-year follow up [7]. All of the cases of recurrence in this study were due to resumed intravenous drug use. The patient cohort may be a reflection of the population at our medical center but also reflects the increase in drug use that has been seen nationally [8]. Substance addiction remains a difficult condition to treat and recurrence is common. At our institution we have a plan to treat patients with endocarditis with a special focus on those patients with a history of IVDU. First, all patients with endocarditis are evaluated by an infectious disease consult to help medically manage them. Second, all patients are treated with intravenous antibiotics for a total of 6 weeks. Third, a referral to substance abuse rehabilitation centers for all patients with a history of IVDU. Fourth, a referral to a primary care physician for all patients to establish general health follow up.

Despite the results of our study the National findings for the surgical management of IE shows that morbidity and mortality for those undergoing surgery remains high. In a large series, 52.2% of patients presenting with IE underwent surgery. In that same series those who underwent surgery had a mortality rate of 21.5%, compared to 15.3% for those who did not undergo surgery [9]. This may reflect the severity and refractive nature of the disease process in patients that are considered for surgery. Furthermore, in another large cohort of patients with IE, surgery was indicated in 53% of patients, but was only performed in 39%. The most common reasons for not moving forward with surgery were deteriorating neurological status and unacceptable operative risk [10]. This is in line with other published studies that report an operative rate of 30–60% [11, 12]. Surgery for IE has become a well-accepted treatment plan despite the high complication rate because surgical intervention may represent the only option for eradication of the valve infection.

The findings of increased CVA in our patient population is a serious complication that has been reported previously. In a 2012 study [13], Kang et al., found that early surgery for patients with IE with large vegetations has shown to be superior to conventional therapy for reducing embolic strokes and death [13]. Moreover, prior studies have confirmed that there is no apparent survival benefit in delaying surgery for IE in patients with stroke [14]. The patient population at the highest risk are those patients with large vegetation on the aortic and mitral valve. This is especially true in patients who have already experienced a stroke from septic emboli. The patients that undergo surgery are not without risk as there is concern for conversion of an ischemic to a hemorrhagic stroke with anticoagulation needed for cardiopulmonary bypass.

As compared to patients who undergo adult cardiac surgery for traditional indications such as coronary artery disease or valvular degeneration, endocarditis patients tend to have longer LOS. The reasons are multifactorial but one key component is the social issues with these patients. They often have difficulty being transferred to rehab centers or lack stable, and safe living environments at home for intravenous antibiotic administration. The consequence of the longer LOS is the increase use of hospital resources and costs plus the patients are put at a higher risk of nosocomial infections.

One mechanism to improve outcomes of patients with endocarditis is early detection and treatment. The recommendations for management of these patients was recently published by Baddour et al. [5]. All patients with suspected endocarditis should have [1] 3 sets of blood cultures drawn from separate sites and an echocardiogram performed expeditiously. Once the organism is identified then antibiotic specific therapy to sterilize vegetations in IE are started. Furthermore, the duration of therapy must be sufficient to ensure complete eradication of microorganisms within vegetations [15].

## Conclusions

The most common complication of this disease process is congestive heart failure, septic shock, embolic events leading to strokes and other neurologic events [12]. This study demonstrates that while the treatment of IE is complex, the morbidity and mortality rates can be minimized to an acceptable level. Additionally, it highlights that the LOS for these patients is often prolonged for both medical and social reasons. The study emphases the important causative role that resumed IVDU has on recurrent IE after surgery.

Taken together, this report adds to the analysis by providing a modern cohort of surgical patients who received care at a large academic medical center. The updated findings from this study should help guide treatment in this problematic patient population.

## Data Availability

All additional data is available upon request.

## Abbreviations

CVA: Cerebral Vascular Accident
IE: Infective Endocarditis
IVDU: Intravenous Drug Use
LOS: Length of Stay
